# Supplementary material for: Splice-Junction-Based Mapping of Alternative Isoforms in the Human Proteome
Source: Cell Rep. Author manuscript; Available in PMC 2020 Jan 15. (PMC6961840; doi:10.1016/j.celrep.2019.11.026)
Supplement: 2 [file NIHMS1546469-supplement-2.zip › DF1/PXD006675/RightAtrium/RightAtrium_36_TPM3_AISEELDHALNDMTSG.pdf]

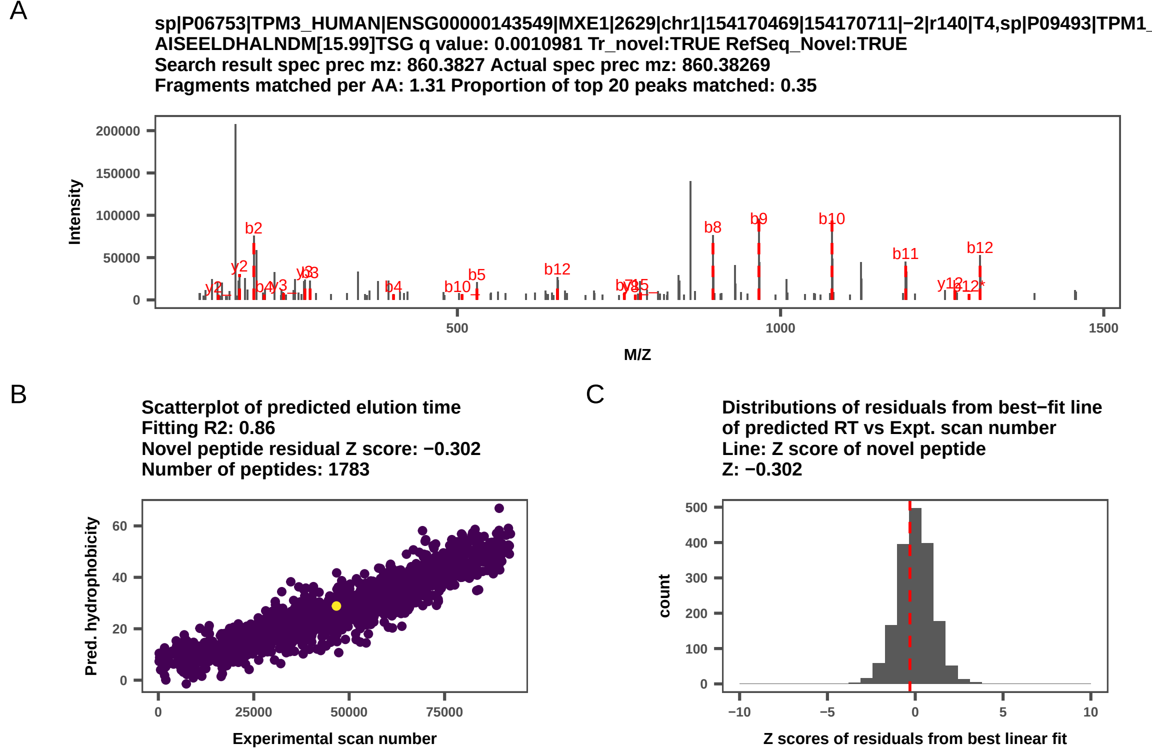

A
sp|P06753|TPM3_HUMAN|ENSG00000143549|MXE1|2629|chr1|154170469|154170711|−2|r140|T4,sp|P09493|TPM1_
AISEELDHALNDM[15.99]TSG q value: 0.0010981 Tr_novel:TRUE RefSeq_Novel:TRUE
Search result spec prec mz: 860.3827 Actual spec prec mz: 860.38269
Fragments matched per AA: 1.31 Proportion of top 20 peaks matched: 0.35
Intensity
0
50000
100000
150000
200000
b2
y2
y2
b4
y3
b3
b4
b10
b5
b12
b8
b9
b10
b11
b12
y12
b12*
M/Z
500
1000
1500
B
Scatterplot of predicted elution time
Fitting R2: 0.86
Novel peptide residual Z score: −0.302
Number of peptides: 1783
Pred. hydrophobicity
0
20
40
60
0
25000
50000
75000
Experimental scan number
C
Distributions of residuals from best−fit line
of predicted RT vs Expt. scan number
Line: Z score of novel peptide
Z: −0.302
count
0
100
200
300
400
500
−10
−5
0
5
10
Z scores of residuals from best linear fit
